# Optimizing polypharmacy management in the elderly: a comprehensive European benchmarking survey and the development of an innovative online benchmarking application

**DOI:** 10.3389/fphar.2023.1254912

**Published:** 2023-10-17

**Authors:** Przemysław Kardas, Alpana Mair, Derek Stewart, Paweł Lewek

**Affiliations:** ^1^ Department of Family Medicine, Medication Adherence Research Centre, Medical University of Lodz, Lodz, Poland; ^2^ Effective Prescribing and Therapeutics Division, Edinburgh Napier University, Edinburgh, United Kingdom; ^3^ College of Pharmacy, QU Health, Qatar University, Doha, Qatar

**Keywords:** polypharmacy, elderly, older adults, chronic conditions, benchmarking, survey, multimorbidity

## Abstract

**Background:** Polypharmacy, defined as the simultaneous use of multiple medications by a patient, is a worldwide problem of rising prevalence. Paving the way for drug interactions, adverse drug reactions and non-adherence, it leads to negative health outcomes, increased use of healthcare services and rising costs. Since it is closely related to multimorbidity, it peaks in older adults. So far, not many polypharmacy management programs in the elderly have been introduced in practice. However, due to the rapid ageing of European societies, there is an urgent need to implement them more widely.

**Objective:** The aim of this study was to benchmark polypharmacy management programs in the elderly available in Europe and creating a dedicated benchmarking application.

**Methods:** It was a cross-sectional study based on an online survey targeting healthcare professionals and other stakeholders across European countries. Data collected in the survey were reused to design an online benchmarking application.

**Results:** As many as 911 respondents from all but two EU countries took part in this study. Out of the survey participants, 496 (54.4%) reported availability of various activities or formal programs targeting polypharmacy in the elderly that were known to them. These programs had multiple goals, of which improved patient safety was indicated as the most common objective (65.1% of the cases). The most typical settings for such programs was primary care (49.4%), with pharmacists and primary care doctors being indicated most often as those providing the programs (61.7% and 35.5% of cases, respectively). Vast majority of programs applied diverse forms of drug reviews. The identified programs were assessed against four predefined dimensions of effectiveness, applicability, scalability and cost-effectiveness. The lowest scores were obtained within the last of these categories, due to unavailability of relevant data. Based on the survey results, a benchmarking application was constructed. It allows for comparing an individual polypharmacy management program targeting the elderly against the other ones, and particularly, against the national and European context.

**Conclusion:** By providing strong evidence, the findings of this study, coupled with the benchmarking application, can prove valuable in aiding clinicians and policymakers in the implementation and expansion of polypharmacy management programs for the elderly.

## 1 Introduction

Polypharmacy is most often referred to as the simultaneous use of multiple medications by a patient to treat their conditions. Still lacking a standard definition, it is usually operationalised as a scenario of concurrent use of five or more prescribed drugs (Kurczewska-Michalak et al., 2021).

Over the last 2 decades, polypharmacy has become a major public health concern, and a subject of multiple scientific publications. The reason for this is twofold. On the one hand, polypharmacy entails a number of profound consequences. Although the correct multidrug treatment in patients with complex medical problems can improve clinical outcomes, quality of life and life expectancy, polypharmacy is also associated with an increased risk of avoidable harm. Of course, first of all this is true in the case of improper use of multiple medicines, i.e., the so called “inappropriate polypharmacy”. Nevertheless, the more drugs are used concurrently, the higher are the chances that polypharmacy becomes inappropriate. Indeed, particularly in older adults polypharmacy leads to increased prevalence and severity of medication-related problems, such as drug interactions, adverse drug reactions and medication errors, some of which are severe enough to result in profound health repercussions or even death. Polypharmacy can also pave the way to medication non-adherence, with up to 50% of community-dwelling older people who receive four or more medications not taking them as prescribed (Franchi et al., 2021). In older adults it leads to occurrence and worsening of geriatric syndromes. Apart from safety issues concerning individual patients, it also has far-reaching public health, social, and economic consequences, which translates into increased use of healthcare services and costs (Fried et al., 2014). Particularly in older adults, it leads to a higher risk of hospitalization and institutionalization, along with greater healthcare expenditures (Maher et al., 2014).

Another reason for the growing interest in polypharmacy is that its frequency has been rising dramatically (Charlesworth et al., 2015; Carmona-Torres et al., 2018; Zhang et al., 2020). A recent analysis proves that within just 5 years, the prevalence of polypharmacy nearly doubled in the United States, and more than doubled in the Netherlands (Oktora et al., 2019). These negative trends are more than certain to continue, as a number of factors responsible for this problem are also on the rise. This is particularly true for ageing and multimorbidity, i.e., the two interlinked factors which are becoming more and more prevalent globally (Guthrie et al., 2015). However, the current paradigm of healthcare, being generally based on fragmented care and single-disease oriented guidelines, seriously increases the chances of multidrug therapy as well. Unfortunately, clinical trials seldom include the elderly and rarely focus on polypharmacy (Giardini et al., 2018). Hence, clinical guidelines only infrequently address the complex nature of multimorbidity and take the patient’s perspective into consideration, prioritize certain conditions or treatments, and consequently, help to reduce the burden of prescribed drugs (Montori et al.; Farmer et al., 2016).

Another indirect consequence of the above-described interrelationships is that both the prevalence and the magnitude of the problem caused by polypharmacy is the greatest in older adults. An analysis of a large European cohort has found polypharmacy to be present in 32.1% of citizens aged 65 years or above (Midão et al.). Recent data from Poland prove that the older the patients, the more prevalent the polypharmacy. In 2019, it affected 42.1% of individuals aged 65+, and 55.0% of those aged 80+ (Kardas et al., 2021a). As many as 19.1% of the national 65+ cohort was subject to chronic polypharmacy, in the vast majority (68.6%) continuing this status for the period of the whole studied year (Kardas et al., 2021b).

There is a variety of tools aimed at reducing inappropriate polypharmacy (Kaufmann et al., 2014). A recent scoping review identified numerous interventions, of which most involved various types of drug reviews based on either implicit (judgement-based) or explicit (item list-based) criteria (Kurczewska-Michalak et al., 2021). However, even those interventions which are simplified by the use of explicit criteria, such as, e.g., STOPP/START, Beers and Medication Appropriateness Index (MAI), and/or supported by the computerised decision support systems, are used infrequently.

In general, polypharmacy management in older adults is underused, and practical implementation of available interventions is very limited. In fact, healthcare professionals are often either unaware of such tools or disregard them as not being user-friendly (Mc Namara et al., 2017). Indeed, application of various forms of drug reviews was reported in only half of the 32 European countries studied (Bulajeva et al., 2014). At the higher level, polypharmacy does not attract much attention of decision makers either. Despite the significance of the problems created by polypharmacy in older adults, this subject is only seldom tackled in a systematic way. An extensive search for polypharmacy guidance documents across Europe has identified only five countries that actually have such instruments targeting older patients (Stewart et al., 2017a).

As a consequence, there is an urgent need to change the current scenario, and reduce the negative impact that polypharmacy has on both individual patients and whole societies. This requirement is even more appealing due to the fact that not only were many tools created, but also several complete interventions were implemented successfully, mostly on the local level. Such interventions need to be identified, and compared, in order to select the best ones, and allow for their multiplication and scaling up. The SIMPATHY Project (Stimulating Innovation Management of Polypharmacy and Adherence in The Elderly), a European scientific collaboration supported by the Horizon 2020 grant, aimed at introducing of relevant system changes that could facilitate implementation of polypharmacy management programs (Mair et al., 2020). To accomplish this objective, SIMPATHY focused on analysing of current healthcare models and practices for management of inappropriate polypharmacy across the EU, as well as stimulating exchange and adoption of the best practices (Mair et al., 2017a). Therefore, the aim of this study, stemming from the SIMPATHY Project, was to benchmark polypharmacy management programs in the elderly available in Europe. In order to increase usability of the obtained results, and stimulate wider implementation of the best practices in real life settings, we also aimed at creating a benchmarking application—an online tool allowing for comparing an individual polypharmacy management program targeting the elderly against other similar solutions, and particularly, against the national and European context.

## 2 Methods

It was a cross-sectional study based on an online survey targeting healthcare professionals and other stakeholders, which aimed at collecting data on practices of polypharmacy management in older adults across European countries, benchmarking the identified programs, and ultimately designing an online benchmarking application. This study was a part of a larger analysis including both patients and professionals, performed within the framework of SIMPATHY project (Mair et al., 2017a). In this paper, however, we report data collected for various types of professional respondents only. In following paragraphs, the methodology of the study is described in more detail, following the STROBE guidelines (von Elm et al., 2007).

### 2.1 Designing the benchmarking survey questionnaire

The benchmarking survey was designed on firm theoretical grounds, constructed under the framework of the SIMPATHY project, which included a systematic literature review (Stewart et al., 2017a), 9 national case studies and reflection over change management mechanisms (McIntosh et al., 2018), and results of the online survey in polypharmacy experts. Detailed analysis of results of all these works allowed for preliminary identification of four major dimensions against which specific strategies of polypharmacy management were to be assessed, i.e., effectiveness, cost-effectiveness, applicability and scalability. Also, an initial list of relevant parameters could be drafted. It covered 170 items, ranging from the high of 54 for “Effectiveness” dimension, to the low of 36 for “Scalability” dimension. In order to reduce this number, a Delphi-like process of fine-tuning of the list of benchmarking parameters has been implemented. In this process, each domain was filtered-out of the least well-matched items so that the number of items in each of the domains did not exceed 25. As a result of five rounds of iterative reduction, the number of the items was reduced to 88. In the next step, the list of the parameters prepared for the questionnaire was validated by external experts. Assuming that validity refers to the appropriateness, meaningfulness and usefulness of a measure for a specific purpose (Jensen, 2003), this process covered aspects of content validity (operational question: are all the dimensions covered?), construct validity (operational question: how well is each of the dimensions covered?), and criterion validity (synonym: predictive validity—to which extent is a measure able to predict important outcomes?). The list of items was presented to a selected number of external polypharmacy experts who were asked to choose up to seven items within each dimension, and rank them from 1 (for top priority) to 7, for each of the two areas of process and outcome. The experts could also propose new items and provide their comments. Based to the results of prioritising of the preselected items, the first version of the survey questionnaire was prepared. It included 42 criteria items. An intensive internal discussion within the SIMPATHY consortium allowed for further fine-tuning of the questionnaire. Its sixth version was approved for piloting in a limited number of stakeholders in preselected SIMPATHY partner countries (Greece, Poland and the United Kingdom), either in the original English version, or local translation. The survey questionnaire was made available in a dedicated online surveying system, with relevant skip options, according to the previous answers given by the respondents. In order to assess the questionnaire itself, all the respondents were directed to the last section, in which they were to give their opinions on its length and content. In total, 40 responses to the pilot survey were obtained. Following discussion, several minor modifications were introduced, and the final English version of the questionnaire was agreed on and approved for use in the benchmarking survey (see Supplementary Appendix S1).

### 2.2 Benchmarking survey fieldwork

The final version of the benchmarking survey questionnaire was translated from English into eight European languages: Dutch, French, German, Greek, Italian, Polish, Portuguese and Swedish. All these versions were made available online at the Survey Monkey website, with relevant skip options provided. Thus, the participant could answer various number of questions, depending on the responses already given. Survey fieldwork was started on 12 June 2016. Diverse methods were used in order to attract the target population which included healthcare providers, members of professional organizations, policymakers, payers, government authorities, and all other kinds of relevant stakeholders. Invitations to take part in the survey were sent via e-mail to a number of individual stakeholders identified in all 28 Member States. A snowball method was also adopted to increase participation in the survey. The SIMPATHY Ambassadors and several collective bodies (such as major professional organisations active in the field of medicine, pharmacy and nursing, as well as patient organisations, *etc.*) were asked to invite other participants. The links to the survey in all the nine language versions were also made available on the SIMPATHY project website.

According to the benchmarking study protocol, a target number of respondents accepted was 560 (i.e. 20 per each out of 28 EU countries, on average). When the number was reached, according to the continuous analysis of both the number and distribution of the respondents, the decision was made to extend the time to collect survey data by 11 September 2016. This was assumed to increase the response rate in underrepresented groups of stakeholders, such as politicians or policymakers.

### 2.3 Statistical analysis of survey data

The survey data collected in the Survey Monkey system were downloaded and saved in separate files created for each of the nine language versions of the survey. Then, after combining all the nine individual files, single master database was set up. Non-English responses were translated into English (based on the English version of the questionnaire, used for the international survey). Relevant variables were created to assess, in a cumulative way, performance of individual programs within each of the four dimensions (effectiveness, applicability, scalability and cost-effectiveness), along with a composite cumulative variable to assess overall performance (for details and relevant thresholds, see Supplementary Appendix S2). Free-text entries were analysed case-by-case and encoded in a cohesive way. Before running the final test, the master database was quality-checked and debugged.

IF≥90% of responses per country indicated lack of a polypharmacy program in that country; such an example was deemed to be “no intervention country”.

Data exploration included descriptive statistics of characteristics of polypharmacy management programs and their analysis with cumulative variables for each of the four dimensions studied, as well as the overall composite measure. In the benchmarking analysis, cumulative data for the country level were compared.

### 2.4 Design of the benchmarking application

Based on the results of the benchmarking survey, an online benchmarking application was created. The application questionnaire was designed to use the original phrasing of the questions of the SIMPATHY Benchmarking survey in order to collect data on performance of individual programs with regard to the four dimensions (effectiveness, applicability, scalability and cost-effectiveness). A graphical interface of the application was designed to produce figures in which characteristics of individual programs were benchmarked against both national and European data.

## 3 Results

### 3.1 Characteristics of the respondents

The total number of 911 responses were collected in the survey. They were obtained from all but two (Luxemburg nor Malta) EU countries (please note that at the time of conducting the survey execution, the United Kingdom was one of the EU Member States). Additionally, 29 responses came from four non-EU European countries (Faroe Islands, Norway, Switzerland and Ukraine), and another 15 from eight non-European countries. More than half of the respondents (52.8%) represented different classes of pharmacists, 12.8% were doctors, whereas 8.9% were nurses, social workers and other healthcare providers. Detailed characteristics of the respondents are presented in Table 1. The distribution of the respondents varied across the countries (e.g., 33.7% of nurse respondents in Poland vs. 0% in both Belgium and Sweden, 75.5% of pharmacist respondents in Belgium vs. 22.7% in Germany, *etc.*).

**TABLE 1:** Characteristics of the respondents of the benchmarking survey.

Countries of the respondents^a^	N	%
EU countries	867	95.2
Austria	6	0.7
Belgium	49	5.4
Bulgaria	1	0.1
Croatia	5	0.5
Cyprus	5	0.5
Czech Republic	6	0.7
Denmark	3	0.3
Estonia	8	0.9
Finland	1	0.1
France	11	1.2
Germany	44	4.8
Greece	52	5.7
Hungary	6	0.7
Ireland	6	0.7
Italy	57	6.3
Latvia	1	0.1
Lithuania	10	1.1
Netherlands	29	3.2
Poland	98	10.8
Portugal	122	13.4
Romania	6	0.7
Slovakia	2	0.2
Slovenia	5	0.5
Spain	41	4.5
Sweden	48	5.3
United Kingdom^b^	245	26.9
• England	140	15.4
• Northern Ireland	11	1.2
• Scotland	84	9.2
• Wales	10	1.1
**Non-EU European countries**	**29**	**3.2**
Faroe Island	1	0.1
Norway	13	1.4
Switzerland	14	1.5
Ukraine	1	0.1
**Other countries**	**15**	**1.6**
Australia	1	0.1
Canada	1	0.1
India	1	0.1
Israel	2	0.2
Malaysia	1	0.1
Palestine	1	0.1
Turkey	2	0.2
United States	6	0.7
**Respondent category**	**N**	**%**
**Doctors, all**	**117**	**12.8**
primary care doctors	54	5.9
geriatricians	25	2.7
outpatient consultant doctors	9	1.0
hospital based doctors	29	3.2
**Pharmacists, all**	**481**	**52.8**
community pharmacists	114	12.5
primary care pharmacists	149	16.4
hospital pharmacists	118	13.0
clinical pharmacists	100	11.0
**Other healthcare professionals, all**	**81**	**8.9**
nurses	63	6.9
social workers	4	0.4
other healthcare providers	14	1.5
**Other stakeholders, all**	**232**	**25.5**
managers, health system managers	37	4.1
policymakers	20	2.2
politicians	8	0.9
healthcare commissioners	7	0.8
healthcare scientists/researchers	99	10.9
education regulators/commissioners	12	1.3
other	49	5.4
**TOTAL NUMBER OF RESPONDENTS**	**911**	**100.0**

a a healthcare professional’s country of work.

b at the time when the survey was conducted, the United Kingdom was a member of the European Union.

### 3.2 Availability and characteristics of the polypharmacy management programs

**FIGURE 1:**
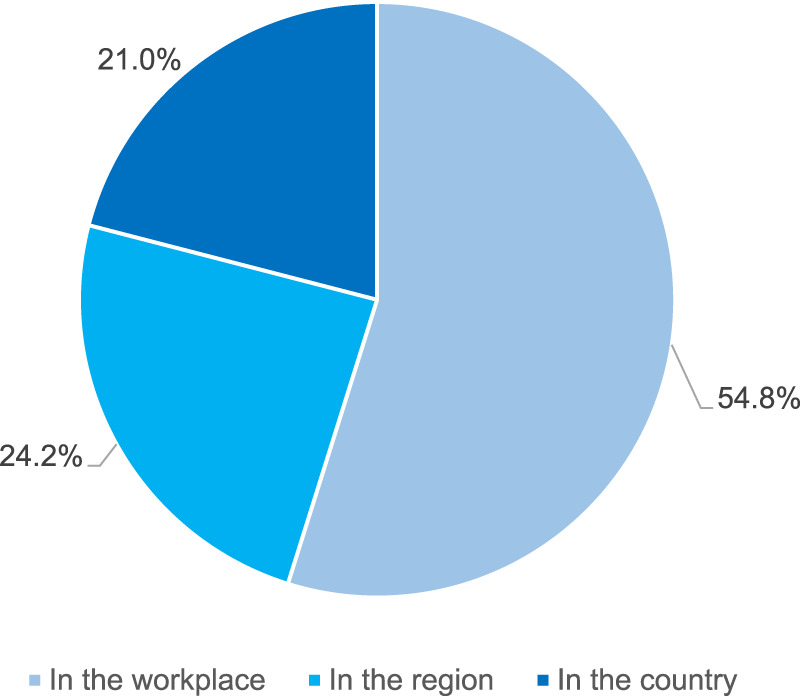
Availability of the polypharmacy management programs in the elderly by the level of their personal contact with the program (N = 496 respondents reporting availability of such programs).

The respondents were asked whether they had any knowledge about an activity or a formal program targeting polypharmacy in the elderly. More than half of them (496, i.e. 54.4%) indicated availability of such programs. In most of the cases, the programs were known to respondents in a direct way, from their workplace (54.8%). However, some of the respondents knew about such programs despite the fact that they did not have any direct contact with them, as they were only available in their region or country only (45.2% in total, for detailed distribution see Figure 1).

**FIGURE 2:**
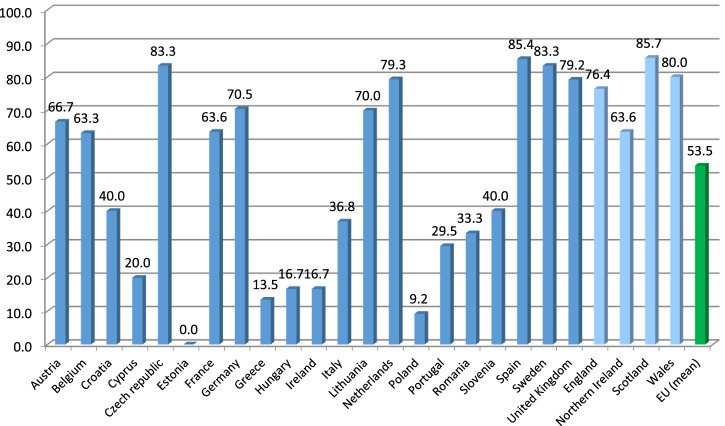
Percentage of the respondents reporting availability of polypharmacy management programs for the elderly across countries of Europe. Note: Countries with at least five responses to the benchmarking survey presented (therefore, Bulgaria, Finland, Latvia, Slovakia and Denmark are not included).

Out of the 26 EU countries from which the responses to the benchmarking survey were collected, polypharmacy management activities or programs for the elderly were reported by all but two countries. On average, more than half of the respondents (53.5%) from the EU countries reported availability of such programs. Due to fulfilling the predefined criterion of programs reported by <10% of the respondents, three EU countries, i.e., Bulgaria, Estonia and Poland, were deemed to be ‘no intervention courtiers’, providing availability of programs in 0%, 0% and 9.2% of their reports, respectively. On the other hand, as many as 14 EU countries reached the level of 50% or more of the respondents reporting availability of polypharmacy management programs for the elderly (these being Austria, Belgium, Czech Republic, Denmark, Finland, France, Germany, Latvia, Lithuania, Netherlands, Slovakia, Spain, Sweden, and the United Kingdom). Among them, seven countries reached a level of 75% or more of the respondents reporting availability of such programs. For detailed distribution of percentages of the respondents reporting availability of polypharmacy management programs for the elderly in their countries, see Figure 2.

Programs known to the respondents had multiple goals often; of these, improved patient safety was provided most often (65.1% of programs). They were followed by programs focused on improved patient health outcomes and reduced medication errors, both reaching the level above 50% of responses (Table 2). Close to this level, there were programs aimed at reduction of hospitalizations number (45.4%), and improved patient adherence to medication (44.6%). The objective of approximately one-third of the programs was cost reduction.

**TABLE 2:** Characteristics of the activities or formal programs targeting polypharmacy in the elderly known to the survey respondents.

Program characteristics	N	%
Major goal of tde program*		
to improve patient safety	323	65.1
to improve patient health outcomes	261	52.6
to reduce medication errors	249	50.2
to reduce the number of hospitalizations	225	45.4
to improve patient adherence to medication	221	44.6
to reduce costs	175	35.3
other goals	34	6.9
don’t know	4	0.8
Program setting*		
primary care	245	49.4
hospital	113	22.8
community pharmacy	103	20.8
hospital pharmacy	48	9.7
other setting	51	10.3
Professionals providing the program*		
pharmacists	306	61.7
GPs (primary care doctors)	176	35.5
other doctors	96	19.4
nurses	76	15.3
other persons	48	9.7
Is the program using teamwork?		
yes: teamwork of doctors + pharmacists	150	30.2
yes: teamwork of doctors + pharmacists + nurses	107	21.6
yes: other patterns of teamwork	47	9.5
no	26	5.2
don't know	19	3.8
Incentives for healthcare professionals providing the program		
it is their contractual responsibility	98	19.8
there are financial incentives for professionals providing the program	88	17.7
it is their legal responsibility	50	10.1
there are other incentives	33	6.7
no incentives	93	18.8
don't know	50	10.1
The program is using*		
Prescription Review	268	54.0
Treatment Review	258	52.0
Clinical Medication Review	233	47.0
A validated medication appropriateness index	108	21.8
Is there a checklist for the intervention designed to help program providers?		
Yes	198	39.9
No	75	15.1
Don’t know	73	14.7
Are electronic patient health records accessible to relevant professionals involved in the program?		
Yes: both to doctors and pharmacists	210	42.3
Yes: only to doctors	31	6.3
Yes: only to pharmacists	9	1.8
No, despite electronic patient health records existing for patients targeted for the program	19	3.8
No, electronic patient health records do not exist for patients targeted for the program	37	7.5
Don’t know	39	7.9

Note: * The respondents could indicate several options at the same time, therefore, numbers do not sum up to the total.

The most typical setting for the programs was primary care (49.4%), with hospitals and community pharmacies being more than twice less common locations (22.8% and 20.8%, respectively). However, pharmacists were indicated as those providing the programs most often (61.7%), with GPs (general practitioners, i.e., primary care doctors) being pointed at much less frequently (35.5%). The programs were often based on teamwork; out of several options, a teamwork of doctors and pharmacists was the most prevalent one (30.2%), followed by a teamwork of doctors, pharmacists and nurses (21.6%). Also, only in some cases there were incentives in place for healthcare professionals providing the program (of which the financial ones were reported for 17.7% of programs only). Techniques applied within the program most often included a prescription review (i.e., a technical review of the list of a patient’s medicines; 54.0%), followed by a treatment review (i.e., a review of medicines with the patient’s full notes; 52.0%) and a clinical medication review (i.e., a face-to-face review of medicines and condition; 47.0%). Other frequently used tools included electronic patient health records accessible to both the doctors and pharmacists involved in the program (42.3%), and a checklist for the intervention designed to help program providers (39.9%).

### 3.3 Effectiveness, applicability, scalability and cost-effectiveness of the polypharmacy management programs

Out of all the 496 respondents, who declared to know about existence of polypharmacy management programs in the elderly, only 148 (29.8%) confirmed awareness of several effectiveness outcome measures of the programs, and were redirected to more detailed questions on this issue. Even fewer respondents (128, i.e. 25.8%) provided answers to the questions assessing various effects of the programs on patients (Table 3). Among them, as many as 42.2% pointed to existence of evidence for a positive effect of the programs on patient satisfaction, whereas 33.6% pointed to evidence of their positive effect on patient health status. Less often, the respondents claimed the programs proved to positively affect patient health-related quality of life (27.3%) and medication adherence (25.0%). Interestingly, none of the respondents pointed to a negative effect of the programs. It is noteworthy, however, that up to half of the respondents answering these questions claimed that data on the effectiveness of these programs were not available, and another 20% chose the “don’t know” option.

**TABLE 3:** Parameter assessing effectiveness of polypharmacy management programs in the elderly.

Opinion on existence of evidence which proves that the program affects	N	%
Patient health status		
Yes—positive effect	43	33.6
Yes—neutral effect	4	3.1
Yes - negative effect	0	0.0
No data available	56	43.8
Don’t know	25	19.5
Patient health-related quality of life		
Yes—positive effect	35	27.3
Yes—neutral effect	4	3.1
Yes - negative effect	0	0.0
No data available	62	48.4
Don’t know	24	18.8
Missing entries	**3**	2.3
Patient satisfaction		
Yes—positive effect	54	42.2
Yes—neutral effect	0	0.0
Yes - negative effect	0	0.0
No data available	47	36.7
Don’t know	25	19.5
Missing entries	**2**	1.6
Patient adherence		
Yes—positive effect	32	25.0
Yes—neutral effect	2	1.6
Yes - negative effect	0	0.0
No data available	63	49.2
Don’t know	28	21.9
Missing entries	**3**	2.3
**TOTAL** ^a^	**128**	100.0

Note: ^a^N = 128 patients who provided valid answers to at least one of four survey questions addressing general effectiveness of the programs.

Parameters assessing the effectiveness of the programs in an objective way were provided very rarely (by 26 respondents only). In their opinion, an average number of drugs reduced after the program had been offered to an individual patient was 1.95+/-1.18 (mean +/-SD). The programs resulted in a mean reduction in medication-related problems by 40.3+/-25.7% on average, as well as reduction in primary care visits due to drug-related problems by 22.2+/-11.5%, and reduction in hospitalisations by 29.2+/-18.8%.

Not too many respondents were aware of the effect the program had on satisfaction among healthcare professionals (those providing the program). It is noteworthy, however, that a positive opinion on this effect was expressed 11 times more often than a neutral one (39.3% vs. 3.6% of the respondents who answered this question). Moreover, literally none of the respondents had a negative opinion.

The respondents who knew about existence of polypharmacy management programs were asked to assess several dimensions of applicability and scalability of these programs. As many as 309 valid answers were provided to this section (corresponding to 62.3% of the programs). From among that number, 56.0% of the respondents declared that the program had been created according to evidence-based (EBM) guidelines (Table 4). In 50.8% of the cases, the respondents reported that the development of skills allowing for multidisciplinary teamwork had been supported in order to help implementation of the program. At first, the support came in the form of educational measures, much less often in the form of financial contributions, via policy initiatives, or through contractual obligations.

**TABLE 4:** Parameters assessing applicability and scalability of polypharmacy management programs in the elderly.

Applicability parameters	N	%
The program was created according to evidence-based (EBM) guidelines		
Yes	173	56.0
No	40	12.9
Don’t know	93	30.1
Missing data	**3**	1.0
Dedicated Information and Communications Technology (ICT) solutions (helping implementation of the program) exist		
Yes	101	32.7
No	128	41.4
Don’t know	70	22.7
Missing data	**10**	3.2
There is a regional or national body coordinating and responsible for the program		
Yes	129	41.7
No	116	37.5
Don’t know	57	18.4
Missing data	**7**	2.3
The development of skills allowing for multidisciplinary teamwork has been supported in order to help implementation of the program		
Yes	157	50.8
No	71	23.0
Don’t know	56	18.1
N/A	22	7.1
Missing data	**3**	1.0
Scalability parameters		
The process of dissemination of guidelines for polypharmacy management and adherence is supported	194	62.8
of which		
• by health authorities	122	39.5
• by professional organisations	120	38.8
• by patients organisations	33	10.7
• by regions	58	18.8
Is not supported	45	14.6
Don’t know	52	16.8
Missing data	**18**	5.8
The program is integrated into undergraduate and/or postgraduate training of practitioners	125	40.5
of which		
• undergraduate training of medical doctors	28	9.1
• postgraduate training of medical doctors	49	15.9
• undergraduate training of pharmacists	56	18.1
• postgraduate training of pharmacists	90	29.1
• undergraduate training of nurses	9	2.9
• postgraduate training of nurses	18	5.8
Is not integrated	81	26.2
Don’t know	83	26.9
Missing data	**20**	6.5
The funding is secured for scaling-up of the program		0.0
Yes	48	15.5
No	132	42.7
Don’t know	112	36.2
Missing data	**17**	5.5
There is an activity taken to raise patient awareness of the program		
Yes	98	31.7
No	115	37.2
Don’t know	74	23.9
Missing data	**22**	7.1

Base: N = 309 participants who provided at least one answer to the relevant question regarding applicability and scalability of polypharmacy management programs; ICT, Information and Communications Technology.

When assessing applicability and scalability of these programs, various enablers were identified. For example, 41.7% of the relevant respondents indicated that there was a regional or national body coordinating and responsible for the program. The presence of dedicated ICT (Information and Communications Technology) solutions that facilitated implementation of the program was indicated by 32.7% of the respondents only. Moreover, the opinions of the respondents on the current level of support the ICT solutions provided to the programs were far from positive, and as many as 60.3% of them assessed them as either somewhat insufficient, or not sufficient.

Several factors may aid effective scalability of the polypharmacy management programs. One of them is undoubtedly the issue of dissemination of guidelines on polypharmacy management. Among the respondents who provided their answers in the applicability and scalability sections of the questionnaire, 62.8% reported that the process of dissemination of guidelines for polypharmacy management had been supported (Table 4). Most often that support came from health authorities (39.5%) and professional organisations (38.8%). In 40.5% of cases the programs were integrated within practitioners’ training, of which most often in undergraduate and postgraduate training of pharmacists (in 29.1%, and 18.1% of respondents, respectively). It seems that there are some activities taken to raise patient awareness of polypharmacy management programs, e.g., through information disseminated in media (31.7% of positive responses). Unfortunately, very infrequently the funding is secured for scaling-up of the programs—this was observed by 15.5% of the respondents only.

Respondents provided wide range of the average percentages of healthcare institutions utilizing electronic prescribing in their country, ranging from 0% to 100.0%. On average, use of electronic prescribing systems was reported very often in primary care settings (91.3%+/-26.2%), and slightly less often in community pharmacies (75.7%+/-38.4%) and hospitals (67.5%+/-37.0%).

A similar pattern was observed for the average percentage of medical institutions trained in implementing such programs within a respondent’s country or region: the highest were the results referring to primary care centres (64.2%+/-38.2%); they were followed by those concerning community pharmacies and hospitals, in which the training was provided twice less often (34.2%+/-38.7%, and 29.6%+/-36.9%, respectively).

Very few respondents were able to provide any evidence concerning the cost-effectiveness of polypharmacy management programs (33 persons, i.e. 6.7% of those who knew about such a program). Only five respondents provided data on the average cost of providing the program for healthcare professional for one patient; these ranged from ‘1 euro per day’ (Switzerland) to 140 euro per drug review (the Netherlands). None of the respondents was able to provide valid estimation of the cost of one quality-adjusted life year (QALY) gained due to the program, and only one—to provide an estimation of the cost of one adverse drug event avoided due to the program (80–180 euro, for Northern Ireland), and one of the cost of one primary healthcare visit avoided due to the program (app. 1,300 euro, for Italy). For the cost of one unplanned hospitalization avoided due to the program, only three estimates were collected; they ranged from 600 to 9,000 euro for Northern Ireland to 2,500 GBP for England. Finally, only five respondents provided estimates of the average net effect of the program per patient (i.e., the difference between saved drug costs and cost of the program per patient), ranging from 35 GBP (Scotland) to 500 euro (Italy).

### 3.4 Benchmarking of the polypharmacy management programs

The parameters of effectiveness, applicability, scalability and cost-effectiveness of the identified programs were assessed according to the predefined criteria, and four cumulative variables were calculated for each program (V_EFFE, V_APPL, V_SCAL, and V_COST, respectively; for details see Methods section).

The results of these calculations show that the identified European programs were most effective within the dimension of applicability, reaching on average 2.57+/-2.07 points. This was followed by dimension of effectiveness (2.31+/-1.89), and scalability (1.80+/-1.59). It is noteworthy that within the dimension of cost-effectiveness, the identified programs reached a very low average score, due to the fact that very few respondents provided estimates of the parameters referring to this dimension. As a consequence, the total average percentage of points collected within all four dimensions for the identified programs, as summarised by the composite measure, reached only 6.81+/-4.51 points (Table 5).

**TABLE 5:** Average benchmarking scores per country. See chapter IV.3 ‘Statistical analysis of survey data’ for details of calculation of cumulative variables (V_EFFE, V_APPL, V_SCAL, and V_COST).

V10 [Q1] which country do you work or live in	No. of responses*	Measure of effectiveness (V_EFFE)	Measure of applicability (V_APPL)	Measure of scalability (V_SCALA)	Measure of cost-effectiveness (V_COST)	Composite measure of benchmarking (V_COMPO)
Belgium	16	1.69	1.44	1.56	0.00	4.69
England	75	2.35	2.21	0.99	0.20	5.75
France	7	2.20	2.15	1.11	0.14	5.60
Germany	24	1.38	1.00	1.17	0.04	3.58
Italy	16	2.56	2.31	1.44	0.19	6.50
Netherlands	18	2.94	3.39	3.22	0.22	9.78
Poland	5	3.40	1.60	1.40	0.40	6.80
Portugal	22	1.27	2.09	1.82	0.00	5.18
Scotland	56	2.68	3.34	1.82	0.21	8.05
Spain	23	2.74	3.52	2.22	0.00	8.48
Sweden	31	2.42	2.90	2.42	0.10	7.84
Other European country	32	2.44	2.94	2.28	0.09	7.75
Other non-European country	25	2.36	2.68	2.40	0.16	7.60
TOTAL*	**350**	**2.31**	**2.57**	**1.80**	**0.13**	**6.82**

Base: 351 individual reports of the respondents who indicated availability of such a program known to them, and provided at least one valid parameter of benchmarking, * number of individual responses providing at least one valid parameter of benchmarking.

### 3.5 Online benchmarking application for polypharmacy management programs

A freely accessible online benchmarking application for polypharmacy management programs has been launched and is available at https://www.zmr.lodz.pl/SIMPATHY-benchmarking-app/.

**FIGURE 3:**
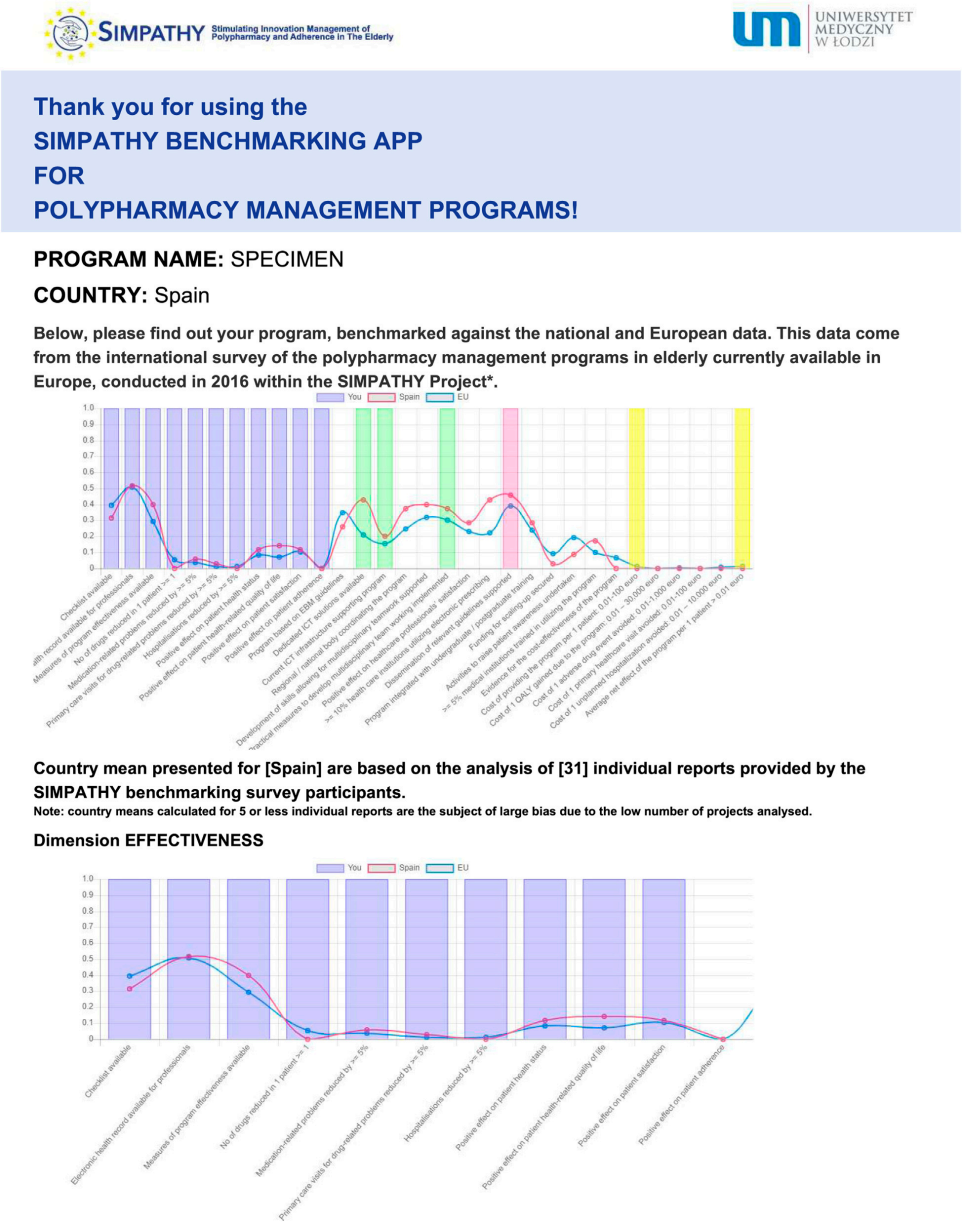
Example of benchmarking of an individual program (specimen) against the trajectory of national, and European means—copy of the report produced by the SIMPATHY benchmarking app available at https://www.zmr.lodz.pl/SIMPATHY-benchmarking-app/(first page presented only).

After the application questionnaire is filled in, a graphical report is produced automatically, along with its printable version (Figure 3). In this report, characteristics of an individual program of polypharmacy management in the elderly are provided with reference to four dimensions, i.e., effectiveness, applicability, scalability and cost-effectiveness. It is also benchmarked to the mean national and European data coming from the SIMPATHY benchmarking survey. Moreover, for the sake of transparency, additional information is provided on the number of responses collected in the benchmarking survey for the country concerned.

## 4 Discussion

This extensive survey included more than 900 respondents representing practically all the EU countries. Most of the survey participants provided a perspective of various classes of healthcare professionals as a majority of them were pharmacists, doctors and nurses. An important finding was big proportion of the study participants—over half of the respondents - reporting availability of different activities or formal programs targeting polypharmacy in the elderly that they were aware of. This is slightly surprising because an extensive search for polypharmacy guidance documents (both those published in scientific journals and made available as grey literature) conducted across Europe within the SIMPATHY Project identified only five countries that actually have such documents targeting older patients (Stewart et al., 2017a). Of course, this might be related to the voluntary nature of the survey, which favoured respondents deeply interested in polypharmacy management in elderly. However, this fact may be also explained by a high number of the identified programs or activities being local initiatives only, and not necessarily having its reflection in the published literature. Indirectly, this emphasizes the value our survey has in terms of illustrating activities otherwise not recorded.

The geographical distribution of the reported programs seems to be far from random. On the one hand, there were countries reporting polypharmacy management programs in the elderly that were available very often (e.g., United Kingdom, Sweden and Spain). On the other hand, only single reports came from some other coutries, and none was obtained from Bulgaria or Estonia. Therefore, the last two, along with Poland with <10% of positive reports, were deemed “no intervention countries”. Considering the fact that the previously mentioned search resulted in finding guidelines for polypharmacy management in the elderly available in selected countries only (i.e., Germany, Spain, Sweden, the Netherlands and United Kingdom) (Stewart et al., 2017a), this uneven distribution is not surprising. The right question to be asked, however, is whether the low number of reported programs in the other countries reflects their unavailability, or rather low awareness of the polypharmacy problem among healthcare professionals, or maybe both. Further studies are required to shed more light on this issue.

It should be stressed that the programs were not incentivised so often, and the use of financial incentives for professionals providing the program was reported by 1/4 of the respondents only. This does not seem to be an optimal approach. Even small financial incentives proved to motivate primary care teams to devote more attention to polypharmacy, which eventually led to significant reductions in related emergency admissions to hospital (Drei et al., 2016). In the light of these data, financial incentives seem to be fully justified even from the economic point of view.

As regards the content of interventions provided within these programs, nearly all of them were based on various forms of drug reviews. In this study, we have not explored any details of these reviews. Therefore, we are lacking information on which tools have been used to conduct them. However, a pragmatic approach to polypharmacy management in the elderly advocates the application of several available explicit criteria-based tools, such as, e.g., STOPP/START, Beers’ criteria, *etc.*, preferably, which may be implemented through a computerized decision-support system (Kurczewska-Michalak et al., 2021).

An interesting finding of our survey is that the respondents assessing the effectiveness of the programs believe that interventions brought several benefits, i.e., they lowered the number of drugs used by a patient, decreased medication-related problems, reduced primary care visits for drug-related problems, and the number of hospitalisations. Similarly, evidence for positive effects of the program has been reported in terms of patient satisfaction, patient health status, patient health-related quality of life, and patient adherence to medication. Unfortunately, the value of these findings is limited due to a low number of respondents providing this data, which was also true for the cost-effectiveness dimension.

As far as the applicability of the programs is concerned, there seems to be a discrepancy between more traditional and more modern tools used to promote them. The programs were often created according to evidence-based guidelines, and the educational measures were implemented to support development of skills facilitating multidisciplinary teamwork for the benefit of the programs. On the other hand, despite the fact that the availability of electronic prescribing was widely reported across the studied countries, the dedicated ICT solutions rather infrequently helped in implementation of the programs, and the majority of the respondents assessed this support to be either somewhat insufficient, or not sufficient. Indeed, computerised systems are extremely useful, yet they may have many disadvantages too. Not only are they often time-consuming but sometimes they also produce dozens of alerts, of which some are of low clinical usefulness, and therefore, subject to ignoring (Knight et al., 2019).

Also, contradictory vectors were observed within the scalability dimension of the identified programs. On the one hand, the process of dissemination of guidelines for polypharmacy management was supported—mostly by health authorities and professional organisations. On the other hand, training in polypharmacy management in the elderly was definitely too rarely integrated into undergraduate and postgraduate education of practitioners. Moreover, activities to raise patient awareness of polypharmacy management programs were probably underused, which is a very common problem (Simões et al., 2022). Finally, the funding for scaling-up of the programs was secured extremely seldom.

To conclude, out of the four predefined dimensions, polypharmacy management programs in the elderly showed the best results within the dimension of applicability, effectiveness and scalability. However, the score for the dimension of cost-effectiveness was significantly lower, in large part due to unavailability of relevant information.

Nevertheless, there are grounds for hope since the respondents who did not know any activity or program targeting polypharmacy management in their workplace, region, or country, expressed their interest in such a program. Indeed, even in countries where polypharmacy management programs do not currently exist, there is a common understanding that polypharmacy is an important issue that needs to be addressed (Stewart et al., 2017b). However, it seems that without some active help, this change will not occur soon—very few found it very probable that such a program could be started in a region/country within the coming 3 years. Therefore, there is a need for further activities aimed at introducing changes in the field of management. To address this need, the SIMPATHY consortium has developed a vision reaching 2030, trying to explore how healthcare management programs can be implemented to improve medication safety and prevent patient harm by addressing the appropriate use of multiple medications (Mair et al., 2017b). Some inspiration can be also found in the Care Pathways, i.e., guiding documents developed by Italian regional health authorities (Dell'Anno et al., 2023).

A recent systematic review identified many cultural and organisational barriers to deprescribing in primary care. As major facilitators of effective deprescribing it listed resources, improved communication, collaboration, patient-centred care and shared decision-making (Doherty et al., 2020). To be effective, the measures to improve the appropriateness of drug use in older people should be implemented across the whole management continuum, from prescription and its acceptance by patient, up to continuous monitoring of adherence and risk-benefit profile (Lunghi et al., 2022).

The results obtained in this study are of high importance. Major lessons learnt from the benchmarking survey of polypharmacy management programs in the elderly conducted across the EU countries are listed in Table 6. Upcoming programs may greatly benefit from these findings. The pattern of future programs should be based on the teamwork of doctors, pharmacists and nurses. It is advisable to make complex interventions, combining medication review with the use of electronic resources (e.g., electronic prescriptions, electronic patient records), which can be implemented thanks to computerised decision-support systems applying one of the validated implicit-criteria based tools. Perhaps, the best results could be obtained with sharing these data among healthcare providers, and overpassing the barriers created by privacy legislation. The use of objective indicators of both effectiveness and cost-effectiveness is more than needed. This is particularly true for the ones generally accepted by the respondents in our survey, such as “reduction in inappropriate prescribing”, “reduction in medication-related problems” and “reduction in hospitalizations due to adverse drug reactions or side effects”, for effectiveness, and “the cost of 1 unplanned hospitalization avoided” for cost-effectiveness. Finally, it would be reasonable to consider financial incentives for institutions and/or individuals providing such programs. When searching for existing gold standards, or designing new schemes, the SIMPATHY benchmarking application might be of great help.

**TABLE 6:** Major lessons learnt due to the SIMPATHY benchmarking survey of polypharmacy management programs in the elderly across the EU countries.

• According to the benchmarking survey results, diverse PMPE are undertaken in most of the EU countries
• PMPE are known to the healthcare professionals
• Most of the PMPE are provided in primary care settings
• PMPE combine patient benefits with cost containment
• There is evidence for effectiveness of PMPE, whereas data on their cost-effectiveness are scarce
• Current ICT infrastructure does not provide effective support for PMPE
• There is a need for better integration of PMPE within practitioners’ undergraduate and postgraduate training
• Wide use of indicators of effectiveness and cost-effectiveness of PMPE is advisable
• The funding for scaling-up of PMPE is not widely available
• Targeted activities within the change management domain are advisable in order to increase the number of PMPE implemented across the European Union

Note: PMPE, polypharmacy management program in the elderly.

On the other hand, one needs to be aware that the data collected in our survey has to be interpreted carefully. Various numbers of the respondents in particular countries, their different background, and underrepresentation of several important stakeholders groups have to be considered. In countries such as Spain and Italy, which have their healthcare systems organised and governed at the regional level, generalization of the findings to the national level is less well supported. In countries such as Spain and Italy, whose healthcare systems are organized and managed at the regional level, generalization of conclusions to the national level should be done with caution. Also, the study has some limitations, related to its voluntary nature and on-line design, namely, the fact that some questions were not answered by many participants. The type of the study did not make it possible to follow up the participants and thus understand the reasons why some of them left the survey before the end. Descriptive nature of this research creates additional limitations. Last but not least, many data—and particularly those related to the cost-effectiveness dimension—may be simply not available for various programs. Moreover, it was not possible to check the quality and reliability of the responses. Therefore, it should be assumed that some of the participants might have given inaccurate answers. However, addressing the survey to the targeted groups of potentially interested stakeholders, we feel that we have minimised the chance of such bias.

These are typical challenges associated with all voluntary surveys, and particularly those made available online. However, this methodology has substantial benefits also, allowing to reach relevant stakeholders living in different geographical locations, and finally, to attract attention of a great number of participants from a large group of countries. In fact, to our knowledge, this was the first study of this kind referring to practical cases and covering the whole Europe. Moreover, approximately 60% of the survey respondents had the opportunity to observe the performance of the projects in their workplace, which means that they shared their own opinions based on their personal experience, rather than other people’s points of view.

## 5 Conclusion

In the coming years, addressing the challenge of polypharmacy in the elderly will be increasingly vital for public health. Consequently, widespread adoption of polypharmacy management programs is an imperative step. To achieve this objective, an evidence-based guidance is essential, aiding clinicians and policymakers in setting realistic drug treatment goals and implementing the most effective strategies available. The findings of this study directly address this need, presenting valuable evidence to guide clinicians and policymakers in the selection and successful implementation of polypharmacy management programs tailored for the elderly. This first-of-its-kind study provides a comprehensive review of polypharmacy management programs for the elderly available across Europe against the criteria of effectiveness, applicability, scalability, and cost-effectiveness. The development of an easy to use benchmarking application adds practical value, encouraging the utilization of these findings. Therefore, the study results, along with the benchmarking application, have the potential to positively affect the trajectory of polypharmacy management, and shape a more effective and sustainable future in elderly care.

## Data Availability

The raw data supporting the conclusion of this article will be made available by the authors, without undue reservation.
