# Supplementary material for: Optimizing polypharmacy management in the elderly: a comprehensive European benchmarking survey and the development of an innovative online benchmarking application
Source: Front Pharmacol. 2023 Oct 17;14:1254912. doi: 10.3389/fphar.2023.1254912 (PMC10616468; doi:10.3389/fphar.2023.1254912)
Supplement: Supplementary file 1 [file DataSheet1.ZIP › Appendixes/SIMPATHY Benchmarking ART_appendix I.pdf]

Introduction

We are inviting you to take part in a survey aiming to capture information about practices in your country that are helping older people taking multiple medicines to gain the best outcomes from their treatment.

What is the purpose of the study?
SIMPATHY (Stimulating Innovation Management of Polypharmacy and Adherence in the Elderly) is a EU project which tackles inappropriate polypharmacy (where people are taking multiple medicines that are not all needed or causing them harm). Polypharmacy is a major health issue which increases the likelihood of adverse effects, impacting significantly on health outcomes and healthcare resources. The final outcome of this project will be tools to enable a strategic approach across the European Union which will guide interventions in polypharmacy and adherence management in the elderly, seeking to improve the health of older people.

Why have I been chosen?
You are one of a number of key stakeholders in your country selected to be invited to participate - those with interests in medicines in older people, such as clinicians, educators, patients, policy makers and politicians.
You may have already provided feedback through other work on SIMPATHY, but we would still value your feedback on the survey.

Do I have to take part?
No, participation in this study is voluntary.

What will happen to me if I take part?
If you decide to take part, you should complete and submit the questionnaire which should take you no more than 20 minutes. The version for patients is even shorter and will take some 5 minutes.

What are the possible benefits of taking part?
The results of the survey will enable you to benchmark your own organisations position across Europe with respect to progress in addressing the issue of polypharmacy and adherence management.

Will my contribution to this study be kept confidential?
Yes, your responses will be completely confidential and any data used will be anonymised.

What will happen to the results of the research study?
We will use the findings to develop tools which will support the management of polypharmacy and adherence throughout the European Union. Next to that, we will produce a short report of the findings, which will be available on the SIMPATHY website, www.simpathy.eu. The findings will also be presented at scientific conferences, and submitted for publication in scientific journals.

What next?
When answering this survey, please consider one programme, intervention or activity well

known to you, aiming to improve the management of polypharmacy, or just rationalising pharmacotherapy. This programme should have a focus on older adults (elderly) but may include other patient groups that have one or more long-term conditions. This might be either local, regional, or national programme. If there are several of such programmes, please assess the one that you are personally involved in. If you are not involved, either directly or indirectly, in any such programme, please assess the one you believe is the best.

What if I am unfamiliar with such a programme?
In some countries there are probably no dedicated activities targeting management of polypharmacy in the elderly at all. If this is the case for your country, you will be provided with some questions related to the design of such a programme, its set up and implementation.

Finally, please be reminded that there are not 'good' nor 'bad' answers. We are keen to understand your perceptions of the programme, and your personal experience.

On behalf of the SIMPATHY project team, thank you for your time

Your data

* 1. Which country do you work (or live if you are a patient) in?

2. Which region within that country do you work (or live if you are a patient) in?

* 3. Who are you (please pick one option):

Characteristics of polypharmacy management programme

4. Is there any activity or formal programme targeting polypharmacy in the elderly known to you (please select all applicable)?

- ☐ a. yes, there is one being employed in my workplace
- ☐ b. yes, there is such an activity in our region
- ☐ c. yes, there is such an activity in our country
- ☐ d. no, there are no such activities known to me
- ☐ e. don't know

Characteristics of polypharmacy management programme (2)

5. What is the major goal of this programme (you may choose several options)?

- ☐ a. to improve patient safety
- ☐ b. to improve patient health outcomes
- ☐ c. to reduce medication errors
- ☐ d. to reduce number of hospitalizations
- ☐ e. to reduce cost
- ☐ f. to improve patient adherence to medication
- ☐ g. don’t know

h. other (please specify)

6. What is the name of this programme?

7. Can you provide the web link to this programme?

8. What is the setting of this programme (please select all applicable)?

- ☐ a. primary care
- ☐ b. hospital
- ☐ c. community pharmacy
- ☐ d. hospital pharmacy

e. other (please specify)

9. Which professionals are providing this programme (please select all applicable)?

- [ ] a. GPs (primary care doctors)
- [ ] b. other doctors
- [ ] c. pharmacists
- [ ] d. nurses

e. other persons – who?

10. Is the programme using teamwork?

- ○ a. Yes: teamwork of doctors + pharmacists
- ○ b. Yes: teamwork of doctors + pharmacists + nurses
- ○ c. Yes: other patterns of teamwork
- ○ d. No
- ○ e. Don't know

11. Are there any incentives for healthcare professionals providing the programme (please select all applicable)?

- [ ] a. Yes – it is their legal responsibility
- [ ] b. Yes – it is their contractual responsibility
- [ ] c. Yes – there are financial incentives for professionals providing the program
- [ ] d. Yes – there are other incentives – please specify below
- [ ] e. No
- [ ] f. Don't know
- [ ] Please specify other incentives

12. Is the programme using (please give your answer to each item):

	Yes	No	Don't know
Prescription Review – a technical review of the list of a patient's medicines	○	○	○
Treatment Review – a review of medicines with the patient's full notes	○	○	○
Clinical Medication Review – a face to face review of medicines and condition	○	○	○
A validated medication appropriateness index	○	○	○

13. Are you personally involved in this programme (please select all applicable)?

- ☐ a. I am personally involved in designing this programme
- ☐ b. I am personally involved in providing this programme
- ☐ c. I am personally involved in supervising this programme
- ☐ d. I am a patient who receives benefit from this programme
- ☐ e. no, I am not personally involved in this programme

f. other (please specify)

EFFECTIVENESS of the programme (1)

14. Is there a checklist for the intervention designed to help programme providers?

- ○ a. Yes
- ○ b. No
- ○ c. Don't know

15. Are electronic patient health records accessible to relevant professionals involved in the programme?

- a. Yes: both to doctors & pharmacists
- b. Yes: only to doctors
- c. Yes: only to pharmacists
- d. No, despite electronic patient health records existing for patients targeted for the programme
- e. No, electronic patient health records do not exist for patients targeted for the programme
- f. Don't know

EFFECTIVENESS of the programme (2)

16. Are you aware if any outcome measures regarding the effectiveness of this programme are known?

- a. Yes
- b. No
- c. Don't know

EFFECTIVENESS of the programme (3)

17. What is the average number of drugs reduced after the programme has been provided to an individual patient (for options b. & c., please input X in relevant box)?

a. number of drugs reduced:

b. Data are unavailable

c. Don't know

18. What is the average percentage reduction in medication-related problems as a result of the programme, e.g. adverse drug reactions (for options b. & c., please input X in relevant box)?

a. average percentage reduction (%):

b. Data are unavailable

c. Don't know

19. What is the average percentage reduction in primary care visits for drug-related problems (e.g. adverse drug reactions, drug therapy adjustments) due to the programme (for options b. & c., please input X in relevant box)?

a. average percentage reduction (%): [ ]

b. Data are unavailable [ ]

c. Don't know [ ]

20. What is the average percentage reduction in hospitalisations due to programme (for options b. & c., please input X in relevant box)?

a. average percentage reduction (%): [ ]

b. Data are unavailable [ ]

c. Don't know [ ]

21. Is there any evidence that the programme affects patient health status?

- a. Yes – positive effect
- b. Yes – neutral effect
- c. Yes - negative effect
- d. No data available
- e. Don't know

22. Is there any evidence that the programme affects patient health-related quality of life?

- a. Yes – positive effect
- b. Yes – neutral effect
- c. Yes – negative effect
- d. No data available
- e. Don't know

23. Is there any evidence that the programme affects patient satisfaction?

- a. Yes – positive effect
- b. Yes – neutral effect
- c. Yes - negative effect
- d. No data available
- e. Don't know

24. Is there any evidence that the programme improves patient adherence?

- a. Yes – positive effect
- b. Yes – neutral effect
- c. Yes - negative effect
- d. No data available
- e. Don't know

APPLICABILITY of the programme

25. Is the programme based on evidence-based (EBM) guidelines?

- a. Yes - please specify below which guideline
- b. No
- c. Don't know

Please specify which guideline

26. Are there any dedicated Information and Communications Technology (ICT) solutions helping implementation of the programme (e.g. electronic polypharmacy management system, clinical decision aid)?

- a. Yes - please specify below what sort of solution?
- b. No
- c. Don't know

Please specify what sort of solution

27. Do you believe that your current ICT infrastructure is supporting a polypharmacy strategy consistently across the different providers / levels of care?

- a. Yes, it is completely sufficient.
- b. It is somewhat sufficient.
- c. It is somewhat insufficient
- d. No, it is not sufficient
- e. Don't know

28. Is there any regional or national body coordinating and responsible for the programme (e.g. ministry of health, local government, etc.)?

- a. Yes – please specify below what sort of body
- b. No
- c. Don't know

please specify what sort of body (give the name)

29. Has the development of skills allowing for multidisciplinary teamwork been supported in order to help implementation of the programme?

- a. Yes
- b. No
- c. Not relevant (programme does not use teamwork)
- d. Don't know

APPLICABILITY of the programme - additional information

30. How has the development of skills allowing for multidisciplinary teamwork been supported in order to help implementation of the programme (please mark all applicable)?

- [ ] a. with educational measures
- [ ] b. with financial measures
- [ ] c. via policy initiatives
- [ ] d. through contractual obligations
- [ ] e. other – please specify below
- [ ] f. don't know
- [ ] Please specify what sort of other means

APPLICABILITY of the programme – Outcome

31. Is there any evidence of the effect of the programme on healthcare professionals' (those providing the programme) satisfaction?

- a. Yes – positive effect
- b. Yes – neutral effect
- c. Yes - negative effect
- d. No data available
- e. Don't know

SCALABILITY of the programme

32. What is the average percentage of health care institutions utilizing electronic prescribing in your country (for option d., please input X in relevant box)?

a. primary care centres	
b. hospital	
c. community pharmacies	
d. don't know	

33. Is the process of dissemination of guidelines for polypharmacy management and adherence supported (please select all applicable)?

- a. Yes – by health authorities
- b. Yes – by professional organisations
- c. Yes – by patients organisations
- d. Yes – by regions
- e. No
- f. Don't know

34. Is the programme integrated within practitioners’ undergraduate and/or postgraduate training (please select all applicable)?

- [ ] a. Yes – undergraduate training of medical doctors
- [ ] b. Yes – postgraduate training of medical doctors
- [ ] c. Yes – undergraduate training of pharmacists
- [ ] d. Yes – postgraduate training of pharmacists
- [ ] e. Yes – undergraduate training of nurses
- [ ] f. Yes – postgraduate training of nurses
- [ ] g. No
- [ ] h. Don't know

35. Is there funding secured for scaling-up of the programme?

- ○ a. Yes
- ○ b. No
- ○ c. Don't know

36. Is there any activity taken to raise patient awareness of the programme (e.g. information in media)?

- ○ a. Yes
- ○ b. No
- ○ c. Don't know

37. What is the average percentage of medical institutions trained in utilizing the programme within your country or region (for option d., please input X in relevant box)?

a. primary care centres ____________________

b. hospitals ____________________

c. community pharmacies ____________________

d. don't know ____________________

COST-EFFECTIVENESS of the programme (1)

38. Are you able to provide any evidence around the cost-effectiveness of this programme?

- ◯ a. Yes
- ◯ b. No but it would be useful information
- ◯ c. No and it would not be useful information
- ◯ c. Don't know

COST-EFFECTIVENESS of the programme (2)

39. What is the average cost of providing the programme for healthcare professional per one patient (for option b. or c., please input X in relevant box)?

a. ... euro	
b. No data are available	
c. Don't know	

40. What is the cost of 1 quality-adjusted life year (QALY) gained due to the programme (for option b. or c., please input X in relevant box)?

a. ... euro	
b. No data are available	
c.Don't know	

41. What is the cost of 1 adverse drug event avoided due to the programme (for option b. or c., please input X in relevant box)?

a. ... euro	
b. No data are available	
c.Don't know	

42. What is the cost of 1 primary healthcare visit avoided due to the programme (for option b. or c., please input X in relevant box)?

a. ... euro	
b. No data are available	
c.Don't know	

43. What is the cost of 1 unplanned hospitalization avoided due to the programme (for option b. or c., please input X in relevant box)?

a. ... euro

b. No data are available

c.Don't know

44. What is the average net effect of the programme per patient (the difference between saved drug costs - cost of the programme per patient) (for option b. or c., please input X in relevant box)?

a. ... euro

b. No data are available

c. Don't know

45. Have you finished your answers in this section?

- Yes

Countries without the programme

46. To what extent is there such a programme needed in your region/country?

- a. very needed
- b. needed
- c. neither needed nor not needed
- d. not needed
- e. not needed at all
- f. don't know

47. How probable is it that such a programme will start in your region/country within the next 3 years?

- a. very probable
- b. probable
- c. neither probable nor improbable
- d. not probable
- e. not probable at all
- f. don't know

48. Have there been any steps made towards the development and implementation of such a programme?

- a. Yes – please specify below what sort of steps
- b. No
- c. Don't know

please specify what sort of steps

49. What you believe would be the best setting for such a programme?

- a. primary care
- b. hospital
- c. community pharmacy
- d. hospital pharmacy

e. other settings - please specify

50. Which professionals should provide this programme (please select all applicable)?

- a. GPs (primary care doctors)
- b. other doctors
- c. pharmacists
- d. nurses

e. other professionals - please specify

51. Should the programme use teamwork?

- a. Yes: teamwork of doctors + pharmacists
- b. Yes: teamwork of doctors + pharmacists + nurses
- c. Yes: other patterns of teamwork
- d. No
- e. Don't know

52. Should there be any incentives for healthcare professionals providing the programme?

- [ ] a. Yes – it should be their legal responsibility
- [ ] b. Yes – it should be their contractual responsibility
- [ ] c. Yes – financial incentives for professionals providing the program should be used
- [ ] d. Yes – other incentives – please specify below
- [ ] e. No
- [ ] f. Don't know
- [ ] Pease specify what sort of other incentives

53. Should the programme use (please give your answer to each item):

	Yes	No	Don't know
Prescription Review – a technical review of the list of a patient's medicines	○	○	○
Treatment Review – a review of medicines with the patient's full notes	○	○	○
Clinical Medication Review – a face to face review of medicines and condition	○	○	○
A validated medication appropriateness index	○	○	○

54. Please mark 'Yes' to continue to the final part of the questionnaire

○ Yes

Patient section

55. Are you completing the survey on behalf of yourself or someone else (a relative, friend or patient group).

○ a. Self
○ b. Elderly relative
○ c. Elderly friend
○ d. Patient group which I represent

56. Do you know of any specific activities or support from your healthcare provider to help patients who are using 5 or more medications to manage the multiple medications they are taking?

- a. Yes
- b. No
- c. Don't know

Patient section (2)

57. Did you or someone you represent ever participate in such an activity (for example a review of your medicines)?

- a. Yes
- b. No
- c. Don't know

Patient section (3)

58. Do you think you or someone you represent is in need of support in managing their multiple medications?

- a. Yes
- b. No
- c. Don't know

Patient section (4)

59. Do you agree that the health, well-being and quality of life of patients who are prescribed multiple medications would benefit (or already benefit) from support from their healthcare provider in managing and understanding their medicines better?

- a. strongly agree
- b. agree
- c. don't know
- d. disagree
- e. strongly disagree

60. Do you agree that providing a healthcare service to help patients to manage multiple medicines has (or would have) a positive benefit on the costs paid by patients for medicines?

- a. strongly agree
- b. agree
- c. don't know
- d. disagree
- e. strongly disagree

61. Please rank in order of importance to you the possible benefits from improved medicines management and support for patients using multiple medications – from 1 for the most important, to 5 for the least important:

	a. Reducing the total number of drugs prescribed for the patient, without reducing the overall beneficial effects
	b. Preventing the unnecessary use of high risk drugs (those with significant side-effects or negative health effects)
	c. Improving quality of life by reducing medication related problems (harmful drug interactions, side-effects)
	d. Avoiding the need to visit your doctor to report problems with medication
	e. Reducing the possibility of needing to be taken to or admitted to hospital due to problems with medication

62. Have you finished your answers in this section?

- Yes

Indicators

63. Please rank these indicators of effectiveness of the programme – from 1 for the most important, to 5 for the least important:

- [ ] a. reduction in the average number of drugs prescribed to an individual patient
- [ ] b. reduction in inappropriate prescribing (i.e. reduction of use of high risk/unnecessary drugs., e.g. benzodiazepines, NSAIDs, etc.)
- [ ] c. reduction in medication-related problems
- [ ] d. reduction in primary care visits for drug-related problems (e.g. adverse drug reactions, drug therapy adjustments)
- [ ] e. reduction in hospitalizations due to adverse drug reactions or side effects

64. Please rank these indicators of cost-effectiveness of the programme – from 1 for the most important, to 5 for the least important:

- [ ] a. the cost of 1 quality-adjusted life year (QALY) gained
- [ ] b. the cost of 1 adverse drug event avoided
- [ ] c. the cost of 1 primary healthcare visit avoided
- [ ] d. the cost of 1 unplanned hospitalization avoided
- [ ] e. the average net effect of the programme per patient (the difference between saved drug costs - cost of th[illegible] programme per patient)

Final questions

65. May we recontact you to retake this survey in 10 months' time to find out how multiple medication management might have progressed in your country? If so, please let us have your e-mail, and we will get back to you.

That's all! Thank you for completing this survey! For more information on SIMPATHY, please visit www.SIMPATHY.eu

PS. Please help us gather as many responses as possible by providing opportunity to take part in this survey to the other European stakeholders. Send this link to your friend: https://www.surveymonkey.com/r/SIMPATHYsurvey
