# Supplementary material for: Optimizing polypharmacy management in the elderly: a comprehensive European benchmarking survey and the development of an innovative online benchmarking application
Source: Front Pharmacol. 2023 Oct 17;14:1254912. doi: 10.3389/fphar.2023.1254912 (PMC10616468; doi:10.3389/fphar.2023.1254912)
Supplement: Supplementary file 1 [file DataSheet1.ZIP › Appendixes/SIMPATHY Benchmarking ART_Appendix II.docx]

## Appendix II

Cumulative variables created to assess the performance of the polypharmacy management programs, and the respective thresholds:

| **Dimension** | **Variable name** | **Description** | **Thresholds & ranges** |
| --- | --- | --- | --- |
| Effectiveness | V_EFFE | Score due to answers to Q14-Q24 | 1 point for each relevant answer: Q14 a, Q15 a, b or c, Q16 a, Q17 a >= 1, Q18 a >=5%, Q19 a >=5%, Q20 a >=5%, Q21 a, Q22 a, Q23 a, Q24 a; with values ranging from 0 to 11 |
| Applicability | V_APPL | Score due to answers to Q25-Q31 | 1 point for each relevant answer: Q25 a, Q26 a, Q27 a or b, Q28 a, Q29 a, Q30 a - e, Q31 a; with values ranging from 0 to 7 |
| Scalability - | V_SCAL | Score due to answers to Q32-Q37 | 1 point for each relevant answer: Q32 any of a-c >= 10%, Q33 a-d, Q34 a - f, Q35 a, Q36 a, Q37 any of a-c >= 5%; with values ranging from 0 to 6 |
| Cost-Effectiveness | V_COST | Score due to answers to Q38-Q44 | 1 point for each relevant answer: Q38 a, Q39 a any value between 0.01 – 100 euro, Q40 a any value between 0.01 – 30,000 euro, Q41 a any value between 0.01 – 1,000 euro, Q42 a any value between 0.01 – 100 euro, Q43 a any value between 0.01 – 10.000 euro, Q44 any value above 0.01 euro; with values ranging from 0 to 7 |
| Overall performance of the program | V_COMPO: | a sum of values for V_EFFE, V_APPL, V_SCAL, and V_COST | values ranging from 0 to 31 |
